# A pilot pragmatic trial of a “what matters most”-based intervention targeting intersectional stigma related to being pregnant and living with HIV in Botswana

**DOI:** 10.1186/s12981-022-00454-3

**Published:** 2022-06-23

**Authors:** Lawrence H. Yang, Evan L. Eschliman, Haitisha Mehta, Supriya Misra, Ohemaa B. Poku, Patlo Entaile, Timothy D. Becker, Tadele Melese, Merrian J. Brooks, Marlene Eisenberg, Melissa A. Stockton, Karen Choe, Danielle Tal, Tingyu Li, Vivian F. Go, Bruce G. Link, Shathani Rampa, Valerie W. Jackson, Gorata D. Manyeagae, Tonya Arscott-Mills, Melody Goodman, Philip R. Opondo, Ari R. Ho-Foster, Michael B. Blank

**Affiliations:** 1 Department of Social and Behavioral Sciences, School of Global Public Health, New York University, 708 Broadway, New York, NY 10003 USA; 2 Department of Epidemiology, Mailman School of Public Health, Columbia University, New York, NY USA; 3 Department of Health, Behavior and Society, Bloomberg School of Public Health, Johns Hopkins University, Baltimore, MD USA; 4 Department of Clinical and Counseling Psychology, Teachers College, Columbia University, New York, NY USA; 5 Department of Public Health, San Francisco State University, San Francisco, CA USA; 6 Division of Gender, Sexuality, and Health, HIV Center for Clinical and Behavioral Studies, Columbia University and New York State Psychiatric Institute, New York, NY USA; 7 Botswana-UPenn Partnership, Gaborone, Botswana; 8 Department of Psychiatry, Icahn School of Medicine at Mount Sinai, New York, NY USA; 9 Department of Obstetrics and Gynaecology, Faculty of Medicine, University of Botswana, Gaborone, Botswana; 10 Department of Pediatrics, Craig Dalsimer Division of Adolescent Medicine, Children’s Hospital of Philadelphia, Philadelphia, PA USA; 11 Department of Psychiatry, Perelman School of Medicine, University of Pennsylvania, Philadelphia, PA USA; 12 Department of Psychiatry, Columbia University Vagelos College of Physicians and Surgeons & New York State Psychiatric Institute, New York, USA; 13 Department of Health Behavior, Gillings School of Global Public Health, University of North Carolina at Chapel Hill, Chapel Hill, USA; 14 School of Public Policy, University of California Riverside, Riverside, CA USA; 15 Department of Sociology, University of California Riverside, Riverside, CA USA; 16 Department of Psychology, University of Botswana, Gaborone, Botswana; 17 Department of Anesthesia, University of California San Francisco, San Francisco, CA USA; 18 Department of Statistics, Faculty of Social Sciences, University of Botswana, Gaborone, Botswana; 19 Department of Pediatrics and Adolescent Health, Faculty of Medicine, University of Botswana, Gaborone, Botswana; 20 Center for Global Health, Perelman School of Medicine, University of Pennsylvania, Philadelphia, PA USA; 21 Department of Biostatistics, School of Global Public Health, New York University, New York, NY USA; 22 Department of Psychiatry, Faculty of Medicine, University of Botswana, Gaborone, Botswana; 23 Office of Research and Graduate Studies, Faculty of Medicine, University of Botswana, Gaborone, Botswana

**Keywords:** Intersectional stigma, Stigma intervention, Pregnant women living with HIV, Culture, Botswana

## Abstract

We conducted a pilot trial of an intervention targeting intersectional stigma related to being pregnant and living with HIV while promoting capabilities for achieving ‘respected motherhood’ (‘what matters most’) in Botswana. A pragmatic design allocated participants to the intervention (N = 44) group and the treatment-as-usual (N = 15) group. An intent-to-treat, difference-in-difference analysis found the intervention group had significant decreases in HIV stigma (*d* = − 1.20; 95% CI − 1.99, − 0.39) and depressive symptoms (*d* = − 1.96; 95% CI − 2.89, − 1.02) from baseline to 4-months postpartum. Some, albeit less pronounced, changes in intersectional stigma were observed, suggesting the importance of structural-level intervention components to reduce intersectional stigma.

**Supplementary Information:**

The online version contains supplementary material available at 10.1186/s12981-022-00454-3.

## Introduction

Pregnant women living with HIV (WLHIV) are particularly vulnerable to intersectional stigma. Intersectional stigma differs from single-axis conceptualizations of stigma by explicitly recognizing how multiple systems of oppression intersect to synergistically compound health inequities [1]. Thus for pregnant WLHIV, the systems of oppression that marginalize the statuses of living with HIV, womanhood, and pregnancy result in intersectional stigma that can negatively impact health and well-being.However, little to no research applies an intersectional intervention approach to the stigma experienced by this population. Additionally, because intersectional stigma is a consequence of structural-level influences, its negative health effects depend on context. This means that intersectional stigma research in contexts where a disproportionate number of pregnant women living with HIV reside, such as sub-Saharan Africa (SSA), is especially needed. An important step in addressing these interlocking systems of oppression is developing interventions that account for participants’ multiple stigmatized statuses (i.e., stigmatized illnesses, social identities, and/or other marginalized statuses).

The intersectional stigma experienced by pregnant WLHIV in SSA harms health and well-being, but few studies have considered these stigmas’ negative effects from an explicitly intersectional perspective. Research in SSA has documented associations between HIV-related stigma in general and poor maternal and infant health outcomes, including nonadherence to antiretroviral therapy (ART) and worsened maternal depression [2]. Studies have also shown that HIV-related stigma in SSA can vary in its negative effects for different sociodemographic groups, such as people of different genders. Gendered conceptions for women link HIV with promiscuity and immorality, and can lead to being blamed for contracting HIV and spreading it to partners, feared and actual abandonment by partners, and community rejection [3]. These gendered manifestations of HIV-related stigma can further intersect with pregnancy, as HIV-related stigma is often elicited during routine antenatal care (e.g., pregnant WLHIV being identified first through routine testing for prevention of mother to child transmission [PMTCT] [4]); further, in addition to rejection by partners and the community, women may experience rejection from clinic staff who may hold beliefs that women with HIV should not become pregnant or become mothers [4].

Yet despite increased recognition of the negative impacts of intersectional stigma on HIV outcomes [5, 6], HIV-related stigma interventions have rarely, if ever, directly targeted intersectional stigma or measured stigma interventions’ effects on intersectional stigma [7, 8]. Intervening on a single stigma likely fails to be responsive to the intricacies of many WLHIV’s lived experiences and the complex consequences of HIV-related intersectional stigma WLHIV may face [9–11]. An intersectional stigma intervention is sorely needed for pregnant WLHIV in Botswana, a country whose HIV prevalence consistently ranks among the world’s highest (i.e., 20.7% of individuals aged 15–49 in 2019 [12]) and where HIV-related stigma has been linked to a significant drop-off in adherence to ART postpartum [13]. We thus addressed this urgent health need by using the “what matters most” (WMM) theory to identify and target an intervention toward the intersectional stigma experienced by pregnant WLHIV in Botswana.

First, WMM was used in formative qualitative research to identify culturally salient ways that intersectional stigma manifests for pregnant WLHIV in Botswana. The WMM theory conceptualizes how stigma is felt most acutely when people are unable to achieve ‘full personhood’ by participating in the activities that ‘matter most’ in their local context [14]. In Botswana, we found achieving full ‘womanhood’ is primarily expressed by being a ‘respected mother.’ Correspondingly, our two-step directed content analysis of 5 focus group discussions and 46 in-depth semi-structured interviews of people with known and unknown HIV status in Botswana using the WMM framework found HIV-related stigma threatens ‘womanhood’ because having HIV elicits perceptions of promiscuity and risks abandonment by partners, thus threatening achievement of ‘womanhood’ that accompanies giving birth. Then, during pregnancy, this stigma further threatens ‘respected motherhood’ by eliciting perceptions of irresponsibility by potentially endangering the baby (i.e., by exposing the baby to HIV) [4].

These findings were then used to develop an intervention designed to counter the specifics of this intersectional stigma experienced by pregnant WLHIV in Botswana. We also conceptualized how achieving WMM (i.e., ‘respected motherhood’) could mitigate stigma. For pregnant WLHIV in Botswana, achieving full ‘womanhood’ can involve bearing a healthy child by mitigating risk for perinatal HIV transmission, including through ART adherence. This conceptualization provided the foundation for the resulting Moving Mothers towards Empowerment (MME) intervention, administered during pregnancy, which consists of 8 group sessions, is co-led by a trained counselor and a peer mother with HIV, and incorporates key empirically-based strategies to reduce stigma (i.e., psychoeducation, cognitive restructuring, coping skills) [15]. Each of these intervention components is targeted toward countering intersectional stigma by promoting the capabilities to achieve WMM among pregnant WLHIV.

We conducted a pilot pragmatic trial to assess whether MME, by bolstering the capabilities associated with ‘respected motherhood,’ targets the intersectional stigma experienced by pregnant WLHIV and may be more effective than interventions that target HIV-related stigma alone. We present data on select implementation outcomes of this novel intervention (i.e., adoption and acceptability), measurement of this intersectional stigma and initial outcome results, focusing on differences in HIV-related stigma, intersectional stigma for WLHIV in Botswana, and psychosocial outcomes (e.g., depressive symptoms) at baseline and 4-months postpartum. Given that maternal prenatal stress has been linked to infant birth outcomes [16], suggesting an indirect relation between HIV stigma and birth outcomes, we also present exploratory infant birth outcomes.

## Methods

We used a pragmatic study design to test our MME intervention against a treatment as usual (TAU) control group in Gaborone, Botswana while also assessing implementation outcomes of adoption and acceptability. Study outcomes for mothers were assessed at baseline during pregnancy and 4-months postpartum, and for infants, at birth.

### Setting, recruitment, and eligibility

We recruited participants from eight high-volume government-run antenatal health facilities operated under the Greater Gaborone District Health Management Team (GGDHMT). Pregnant women are routinely tested for HIV as part of free antenatal care (ANC) in Botswana for PMTCT. Six of eight clinics were geographically proximate with a high frequency of eligible participants, which enabled forming intervention groups across clinics at a nearby location.

We included pregnant women who were identified as having HIV, were registered to receive ANC at the identified clinics, and met these inclusion criteria: (a) 18–45 years of age, (b) Botswana citizen, (c) English or Setswana speaking, and (d) no more than 28 weeks pregnant at time of recruitment to allow sufficient time to participate in the intervention before delivery, if allocated so. Women who experienced a miscarriage before 28 weeks were excluded from the study. In addition, women who were unavailable to attend the full intervention before week 36 were only considered for allocation to the control group. Although women were not required to be on ART at enrollment, they generally were expected to be receiving ART during pregnancy per national HIV treatment guidelines.

### Measures

*Stigma* was measured using two validated scales: (1) the Berger HIV Stigma Scale (40 items; α = 0.96), which has four subscales of personalized (i.e., internalized) stigma (18 items; α = 0.95), disclosure concerns (10 items; α = 0.89), negative self-image (13 items; α = 0.90), and concern with public attitudes (20 items; α = 0.94); and (2) to assess intersectional stigma of HIV and ‘womanhood’ in Botswana, our WMM Cultural Stigma Scale for WLHIV in Botswana (WMM-WLHIV-BW) [17], which has two subscales that measure (i) how culture ‘shapes’ stigma by threatening ‘womanhood’ in intersection with HIV (e.g., via perceived promiscuity) (10 items; α = 0.94) and (ii) how culture ‘protects’ against stigma in that achieving capabilities of ‘respected womanhood’ could mitigate against HIV stigma (10 items; α = 0.91). *Other psychosocial outcomes* measured are: (i) depressive symptoms, measured by the Center for Epidemiological Studies Depression Scale (CES-D; 20 items; α = 0.88); (ii) post-traumatic stress disorder (PTSD) symptoms, measured by an abridged version of the Post-Traumatic Checklist for DSM-5 (PCL-5; 8 items; α = 0.93); (iii) social functioning, measured by the World Health Organization Disability Assessment Schedule 2.0 (WHODAS-12; 12 items; α = 0.82); (iv) quality of life, measured by the Patient-Reported Outcomes Measurement Information System (PROMIS) Ability to Participate in Social Roles and Activities Short Form scale (8 items; α = 0.83); and (v) perceived availability of social support measured via rating confidence that, if needed, adequate support would be available across six different domains (6 items; α = 0.84). *Infant birth outcomes* were taken from hospital records, and included the infant’s birth weight (grams), gestational age (number of weeks as determined by the clinician), and APGAR scores at 1 and 5 min. *Implementation outcomes* were assessed by what percentage of participants recruited into the intervention condition attended ≥ 1 session (i.e., adoption; [18]) and of these participants, what percentage attended ≥ 3 additional sessions (i.e., acceptability; [18]). All scales are described in more detail in the study protocol [15].

### Condition assignment and blinding

Pragmatic challenges of recruiting pregnant women during the first 28 weeks of pregnancy made randomization infeasible. Instead, eligible participants were assigned to receive MME or TAU based on their recruitment timing; i.e., based on the feasibility for them to complete eight sessions before week 36 of their pregnancy. Based on recruitment timing, assignment was made solely by staff (i.e., participants did not have any role). After consent was acquired, sessions (~ 90 min) were scheduled to run at least once per week and sometimes twice per week (based on each group’s preference) for a total of 8 sessions. Owing to delays in recruitment related to the COVID-19 pandemic, we further assigned participants to the intervention vs. control groups using a 3:1 ratio to maintain a sufficient sample to detect between-group differences (see “Statistical analysis”). Participants were reimbursed for transportation costs for MME sessions and assessments. Only participants and study staff administering the intervention were aware of treatment allocation; all other team members were blinded during data collection. The biostatistician was unblinded during data analysis.

### Intervention

The MME intervention (‘*Mme’* is a Setswana term for a ‘respected woman’), described in detail in the study protocol [15], is co-led by a trained mental health counselor and a peer mother with HIV. In addition to targeting generalized HIV stigma, the intervention addresses the intersectional aspects of stigma experienced by pregnant WLHIV in Botswana by tailoring key evidence-based intervention components toward bolstering the capabilities that signify ‘respected motherhood’ for this group: (1) psychoeducation [e.g., achieving “respected motherhood” by adhering to ART and other care pre- and postpartum; additional psychoeducation could also take place towards intersectional stigma by defining what it is, how it affects pregnant WLHIV in Botswana, and ways to counter it (see Table 1)]; (2) cognitive restructuring to challenge stereotypes of pregnant women identified with HIV (e.g., reframing ART adherence postpartum as enacting ‘respected motherhood’ to counter stereotypes of being unfit to care for the baby); and (3) enhancing coping (e.g., promoting safe disclosure of HIV status to facilitate ART adherence postpartum, thus fulfilling maternal duties and augmenting community support). Topics covered by the interventions’ sessions and their accompanying components are provided in Table 1. The intervention concludes with the bestowal of a shawl during the final session (i.e., a practice that typically occurs when women marry and are ready to bear children). All women who attended at least one prior session are invited to attend the final session. In addition to reducing stigma via more general mechanisms, helping pregnant mothers with HIV enact these values that “matter most” could help protect against intersectional stigma.

**Table 1:** Mothers moving towards empowerment intervention session topics and stigma intervention component(s) included in each session

Session	Topic	Psychoeducation^a,b^	Cognitive restructuring	Enhancing coping
1	Introduction and defining stigma	X^a^		X
2	Common myths and facts about hiv transmission	X^b^		X
3	Common myths and facts about prevention of mother-to-child transmission (PMTCT)	X^b^		
4	The road to self-acceptance and freedom: coping strategies	X^a^	X	X
5	Coping with discrimination: social support and self-disclosure		X	X
6	The road to self-acceptance and freedom part 2: automatic thoughts		X	X
7	Post-natal care, relapse prevention	X^b^		
8	What matters most, review and graduation	X^a^	X	X

X = included; ^a^Psychoeducation about stigma; ^b^Psychoeducation about HIV/ART

### Treatment as usual

Control participants received TAU, which includes free ART and ANC. They were also assessed on all primary outcomes at baseline and 4-months postpartum. No participants assigned to the TAU condition attended any intervention sessions.

### Ethics

The University of Botswana Institutional Review Board (IRB), the Ministry of Health and Wellness' Health Research and Development Committee of Botswana IRB, the Princess Marina Hospital IRB, the University of Pennsylvania IRB, the New York University IRB, and the Greater Gaborone District Health Management Team approved the trial, which is registered at http://www.clinicaltrials.gov (NCT03698981).

### Statistical analysis

We conducted a difference-in-difference intent-to-treat analysis to compare the intervention and control group from baseline to 4-months postpartum. Given the small sample size, nonparametric tests were used to examine differences between groups. For each woman’s outcome (e.g., stigma, depression, social support) the difference between each participant’s 4-month postpartum and baseline values were calculated. These differences were compared across groups (intervention, control) using a Wilcoxon Rank-Sum test with *p*-values calculated using exact probabilities. Exploratory infant outcomes (e.g., birth weight, gestational age) are assessed at a single time point; birth weights were also converted to weight-for-gestational age z-scores using the INTERGROWTH-21st anthropometric software [19]. Difference between groups are examined using the nonparametric Wilcoxon Rank-Sum test with *p*-values calculated using exact probabilities. Analyses are based on complete data for each outcome which may result in different sample sizes. The mean point estimates for each outcome were used to estimate effect size. Both Cohen’s *d* and Hedge’s *g* (adjusting for different samples sizes in the intervention and control groups) were calculated along with 95% confidence intervals. Statistical analyses were conducted in Stata 16 and statistical significance is assessed as p < 0.05.

### Sample size and power

Given the small sample size we used nonparametric test statistics and exact probabilities to calculate p-values used to make inferences between groups. We calculated power *pre* and post hoc*. *Post hoc power was calculated using Power Analysis and Sample Size Software, based on the Wilcoxon Rank-Sum test. We estimate power based on the smallest sample sizes in our analysis. Group sample sizes of 8 and 25 achieve > 80% power to detect a difference in means between the intervention and control group > 0.2 (small effect size) using a two-sided Wilcoxon Rank-Sum when the significance level (alpha) of the test is 0.05.

## Results

### Participant characteristics

A total of 59 women were enrolled, with 15 allocated to the TAU control group and 44 allocated to the MME intervention group. Participant characteristics overall, and separated by study arm, are presented in Table 2. The mean age at baseline was 27.8 years (SD = 6.1). At baseline, 69% (*n* = 41) of all participants reported having completed at least the equivalent of high school education, 41% (*n* = 24) of participants overall were employed, and the average annual family income of the participants who reported income (*n* = 31) was about BWP 5,275 (~ 472 USD). Additionally, 34% (*n* = 20) of participants overall were currently in a long-term relationship. When examining differences between intervention and control groups across demographic characteristics, intervention group participants appeared to be more likely to not currently be in a long-term relationship (55%) when compared with TAU group participants (20%).

**Table 2:** Baseline characteristics of women enrolled in the Mothers Moving towards Empowerment trial

Characteristic	Overall			Control			Intervention			*p*-value**
	n	Mean (SD)/%*	Median	n	Mean (SD)/%*	Median	n	Mean (SD)/%*	Median	
Number of women enrolled	59	100	–	15	25.4	–	44	74.6	–	–
Age	58	27.8 (6.1)	27	15	27.4 (6.8)	30	43	28 (5.9)	27	0.619
Family income (2018)***	31	5275.5 (7010.4)	2000	8	6062.5 (8064.2)	2500	23	5001.7 (6784)	2000	0.885
Number of people in household	56	4.0 (3.1)	3	15	4.1 (3.5)	3	41	4.0 (3.0)	3	0.713
Relationship status										0.017
Currently in long-term relationship	20	33.9		9	60.0		11	25.0		
Not currently in long-term relationship	26	44.1		3	20.0		23	55.3		
Sexual orientation										0.250
Heterosexual/straight	51	86.4		12	80.0		39	88.6		
Bisexual	1	1.7		1	6.7		0	0.0		
Education										0.354
Completed high school	41	69.5		12	80.0		29	65.9		
Did not complete high school	18	30.5		3	20.0		15	34.1		
Employment										0.756
Employed	24	40.7		7	46.7		17	38.6		
Not employed	31	52.5		7	46.7		24	54.6		
Financial strain										0.507
Short of money 1 or more times/month	15	25.4		5	33.3		10	22.7		
Short of money less than once/month	42	71.2		10	66.7		32	72.7		
Living situation										0.624
Own or rent home/apartment	45	76.3		12	80.0		33	75.0		
Staying at home of family member(s)	9	15.3		3	20.0		6	13.6		
Staying at home of friend(s)/other	3	5.1		0	0.0		3	6.8		

^*^Percentages may not sum to 100 due to missing data; percentages were calculated with denominators of the total n for each group (i.e., 59 for overall, 15 for control, 44 for intervention)

^**^Continuous p-values are based on the Wilcoxon Rank-sum test and exact probabilities. Categorical p-values are based on Fisher's exact test

^***^Documented in Botswana pula (BWP)

Regarding implementation outcomes, intervention adoption was moderate; 58% of eligible participants attended ≥ 1 session. Intervention acceptability was high; once participants attended at least one session, most (75%) stayed in the intervention (attended ≥ 3 additional sessions, $\overline{x}$  = 5.2 sessions).

For outcome variables, the intervention group had significantly higher Berger HIV-related stigma (88.0 vs. 68.7, *p* = 0.001) and depressive symptom scores (27.9 vs. 17.7, *p* = 0.020) than the control group at baseline. All other measured outcome variables were comparable between groups at baseline.

Due to participant dropout at 4-months postpartum and missing values from scales, a total of 46 participants were included in the difference-in-difference analysis, with 12 in the control group and 34 in the intervention group.

### Stigma and psychosocial outcomes

Stigma and psychosocial outcomes among women enrolled in the MME trial, compared by study arm, are presented in Table 3. The intervention group had significant decreases between baseline and 4-months postpartum for HIV-related stigma (Cohen’s *d* = − 1.20; 95% CI − 1.99, − 0.39). The control group had corresponding, non-significant increases in HIV-related stigma (Table 3). The significant decrease in HIV-related stigma scores for the intervention group was seen across all four subscales of the Berger HIV Stigma Scale: personalized stigma (*d* = − 0.86; 95% CI − 1.59, − 0.13), disclosure concerns (*d* = − 1.03; 95% CI − 1.72, − 0.33), negative self-image (*d* = − 1.07; 95% CI − 1.79, − 0.34), and concern with public attitudes (*d* = − 0.93; 95% CI − 1.66, − 0.19). Moderate effect sizes were observed in expected directions for the intersectional stigma scale (WMM-WLHIV-BW) on both its subscales of “Cultural Factors Shape Stigma” (*d* = − 0.59; 95% CI − 1.26, 0.08), and “Cultural Capabilities Protect against Stigma” (*d* = 0.50; 95% CI − 0.17, 1.16). However, the differences between the two groups in differences for these two subscales were not significant at a 0.05 significance level.

**Table 3:** Outcomes of women enrolled in the Mothers Moving towards Empowerment trial

Scale	Control			Intervention			Difference between groups			Effect size					
	n	Mean	Median	n	Mean	Median	Mean	Median	*p*-value^a^	Cohen’s *d*	95% CI		Hedges’s *g*	95% CI	
Stigma outcomes (differences between 4 months postpartum and baseline)															
Berger HIV Stigma Scale	9	14.6	18.0	29	− 14.8	− 15.0	− 29.4	− 33.0	< 0.01	− 1.20	− 1.99	− 0.39	− 1.17	− 1.94	− 0.38
Personalized stigma	10	7.7	7.5	33	− 3.7	− 3.0	− 11.4	− 10.5	0.03	− 0.86	− 1.59	− 0.13	− 0.85	− 1.56	− 0.13
Disclosure concerns	12	1.0	− 0.5	33	− 6.3	− 6.0	− 7.3	− 5.5	< 0.01	− 1.03	− 1.72	− 0.33	− 1.02	− 1.69	− 0.33
Negative self-image	11	2.8	3.0	32	− 6.5	− 6.5	− 9.3	− 9.5	< 0.01	− 1.07	− 1.79	− 0.34	− 1.05	− 1.75	− 0.34
Concern with public attitudes	10	7.9	10.0	33	− 5.5	− 6.0	− 13.4	− 16.0	0.01	− 0.93	− 1.66	− 0.19	− 0.91	− 1.63	− 0.18
WMM-WLHIV-BW—culture shapes	12	7.7	7.0	34	0.7	− 1.0	− 7.0	− 8.0	0.09	− 0.59	− 1.26	0.08	− 0.58	− 1.24	0.08
WMM-WLHIV-BW—culture protects	12	− 2.8	− 0.5	34	1.6	0.5	4.4	1.0	0.22	0.50	− 0.17	1.16	0.49	− 0.17	1.14
Other psychosocial outcomes (differences between 4 months postpartum and baseline)															
Depressive symptoms (CES-D)	8	8.1	7.5	26	− 15.9	− 18.0	− 24.0	− 25.5	< 0.01	− 1.63	− 2.51	− 0.74	− 1.59	− 2.45	− 0.72
PTSD symptoms (PCL-5)	12	0.6	0.0	33	− 4.7	− 2.0	− 5.3	− 2.0	0.28	− 0.54	− 1.21	0.13	− 0.53	− 1.19	0.13
Social functioning (WHODAS-12)	12	− 3.5	− 0.5	33	− 6.9	− 5.0	− 3.4	− 4.5	0.12	− 0.38	− 1.04	0.29	− 0.37	− 1.02	0.29
Quality of life (PROMIS-8)	12	1.4	2.0	33	4.3	4.0	2.9	2.0	0.42	0.28	− 0.38	0.94	0.28	− 0.38	0.93
Social support (6 items)	12	− 5.3	− 4.0	34	1.6	2.5	6.9	6.5	0.10	0.67	− 0.01	1.34	0.66	− 0.49	0.81

All effect sizes are based on mean comparison

*WMM-WLHIV-BW* what matters most cultural stigma scale for women living with HIV in Botswana

^a^*p*-values are based on Wilcoxon rank-sum test using exact probabilities (instead of normal approximation)

Regarding psychosocial outcomes, a large effect size was observed for depressive symptoms (*d* = − 1.63; 95% CI − 2.51, − 0.74). There were moderate effect sizes observed for PTSD symptoms (*d* = − 0.54; 95% CI − 1.21, 0.13) and social support (*d* = 0.67; 95% CI − 0.01, 1.34). Effect sizes for social functioning and quality of life were modest (*d* = − 0.38 and *d* = 0.28, respectively) but in expected directions.

### Exploratory infant birth outcomes

Exploratory birth outcomes, compared by study arm, are presented in Table 4. The two sets of twins born to women enrolled in the trial are excluded from analyses. Infants born to mothers from the intervention group showed statistically significantly higher gestational age at time of birth (mean = 39.7 weeks) than the control group (mean = 38.2 weeks) (*d* = 1.05; 95% CI 0.34, 1.74). Birth weight showed a significantly greater weight for infants born to mothers in the intervention group (3234.7 g) than the control group (2913.3 g) (*d* = 0.65; 95% CI − 0.04, 1.32). However, no significant difference in weight-for-gestational age z-scores between groups was observed. APGAR scores were comparable between groups. Additional data on overall infant characteristics are provided in Additional file 1.

**Table 4:** Exploratory infant birth outcomes of the Moving Mothers towards Empowerment trial

Measurement	Control			Intervention			Difference between groups			Effect size		
	n	Mean	Median	n	Mean	Median	Mean	Median	*p*-value^a^	Cohen’s *d*^b^	95% CI	
Birth weight (overall, grams)	12	2913.3	2877.5	32	3234.7	3115.0	321.4	237.5	0.03	0.65	− 0.04	1.32
Males (grams)^	7	3070.7	3000	13	3145.8	3035	75.1	35.0	0.86	0.20	− 0.72	1.12
Females (grams)^	4	2783.8	2767.5	12	3362.5	3370	578.7	602.5	0.03	0.89	− 0.31	2.05
Gestational age (overall, weeks)	12	38.2	38.0	32	39.7	40.0	1.5	2.0	0.01	1.05	0.34	1.74
Males (weeks)^	7	38.6	38.0	13	39.7	40.0	1.1	2.0	0.26	0.64	− 0.31	1.58
Females (weeks)^	4	38.0	38.0	12	39.7	40.0	1.7	2.0	0.10	1.21	− 0.03	2.40
Weight-for-gestational age (z-score)^^&^	11	− 0.45	− 0.51	25	− 0.16	− 0.06	0.29	0.45	0.38	0.24	− 0.47	0.95
Males (z-score)^	7	− 0.34	− 0.26	13	− 0.51	− 0.26	− 0.17	0.0	0.66	− 0.21	− 1.13	0.72
Females (z-score)^	4	− 0.64	− 0.58	12	0.22	0.21	0.86	0.79	0.10	0.59	− 0.57	1.74
APGAR 1 min	12	8.8	9.0	28	8.7	9.0	− 0.1	0.0	0.61	− 0.31	− 0.98	0.38
Males^	7	9.0	9.0	10	9.0	9.0	0	0	0.99	–	–	–
Females^	4	8.5	8.5	11	8.6	9.0	0.1	0.5	0.99	0.26	− 0.89	1.40
APGAR 5 min	12	9.8	10.0	28	9.6	10.0	− 0.2	0.0	0.82	− 0.20	− 0.88	0.48
Males^	7	9.9	10.0	10	10.0	10.0	0.1	0	0.82	0.60	− 0.40	1.58
Females^	4	9.5	9.5	11	9.5	10.0	0	0.5	0.99	0.08	− 1.06	1.23

Data presented are on singletons only (i.e., two sets of twins were excluded from these analyses). Additional overall infant characteristics are presented in Additional file 1

^a^*p*-values are based on Wilcoxon rank-sum test using exact probabilities (instead of normal approximation)

^b^Calculated using groups’ means from a single time point (i.e., at birth)

^Among singletons with documented sex

^&^Sex and birthweight standardized z-scores determined from study biometry using INTERGROWTH-21st standards, accessed 8 May 2022 from URL: http://intergrowth21.ndog.ox.ac.uk/

## Discussion

Via the WMM theory, we conceptualized how WMM manifests at the intersection of interlocking marginalized statuses of being a woman, pregnant, and living with HIV in Botswana, and how capabilities that “matter most” might be promoted to reduce stigma. These WMM-based insights were used to adapt stigma intervention components to develop a multi-component stigma intervention to specifically counter the intersectional stigma experienced by pregnant women living with HIV in Botswana. Our study also incorporated a new, validated measure of intersectional stigma appropriate for this population (i.e., the WMM-WLHIV-BW; [17])*.* Initial implementation outcomes indicated that intervention adoption was moderate, with 58% of eligible participants attending one or more sessions; further, of those attending at least one session, the vast majority (75%) attended ≥ 3 additional sessions, showing good intervention acceptability. Results from our pilot pragmatic trial indicate preliminary effectiveness for the MME intervention, with significant decreases in HIV-related stigma and depressive symptoms at 4-months postpartum compared with concurrent changes in these outcomes in the control group. Further, while not statistically significant, our intervention also showed moderate effect size changes in expected directions for the two WMM subscales assessing intersectional stigma, thus signaling some, albeit less pronounced, changes in intersectional aspects of gender and HIV stigma in the context of Botswana.

This study comprises a significant step forward for intersectional stigma interventions to address the vulnerability elicited at the intersections of multiple systems of oppression, which have been neglected by HIV stigma interventions to date [7]. To our knowledge, this stigma intervention is one of the first to specifically target intersectional stigma among pregnant women into the postpartum period in SSA and globally. Interventions to reduce HIV-related stigma in general in low- and middle-income countries have mixed effectiveness, with one systematic review finding a range of effect sizes for changes in self-stigma, with only 4 studies reporting a magnitude of *d* > 1.0 out of the 14 studies showing significant reductions in self-stigma [7]. It is possible that our relatively more intensive WMM-based adaptation process helped elicit significant stigma reduction at a magnitude only infrequently achieved by similar interventions. At least some of this reduction in stigma may also be due to the incorporation of peer co-facilitators who have experienced the same intersectional stigma (i.e., are mothers living with HIV) and who had shown the ability to learn and communicate intervention principles, as well as demonstrated the ability to apply these to their own lives during an intensive 3-day training on the intervention curriculum undertaken prior to the trial [15]. Via their prior personal experience, these peer co-facilitators were able to model resilience against stigma and preservation of ‘personhood,’ including helping participants strategize how to safely disclose their HIV status to supportive others.

Our results also indicate that our intervention appears to have changed generalized HIV stigma somewhat moreso than intersectional stigma for this group. Changes in generalized stigma were consistent across four HIV stigma subscales with comparably large effect sizes (*d* = − 0.84 to − 1.07), suggesting that HIV stigma was most readily countered across domains via our MME intervention. That intersectional stigma subscales showed somewhat less change (magnitude of *d* = 0.50 to 0.59) despite our intervention’s deliberate focus on the intersectional vulnerabilities experienced by this group may illustrate the inherent complexity in intervening on intersectional stigma; intersectional stigma interventions would ideally also address interlocking and reinforcing structures that give rise to oppression in addition to mitigating stigma on the individual level. Fully addressing intersectional stigma in our target group may thus require additional focus on relevant organizational and institutional levels (e.g., ANC clinic structures and policies) to address the structural mechanisms by which intersectional stigma is reinforced.

### Limitations

Our study has several limitations. First, whereas societal change is needed to gradually decrease harmful rigidity in gender roles and reduce inequitable power dynamics, we believe that achieving capabilities that “matter most” is particularly valuable when these are participant-identified [4]. Of note, the intervention assists participants in making use of these core values when beneficial and when they are often experienced as paramount (i.e., during pregnancy). Further, in addition to encompassing the capabilities of women at a particular time and place, WMM is future-oriented. By structuring our WMM-based stigma intervention around the ways in which pregnant women with HIV can achieve what people in Botswana currently perceive as “what matters most” (i.e., “being a respected mother”), women with HIV can then gain the capacity and motivation to further evolve these capabilities via taking on other roles [20]. Next, given the small sample, results should be considered preliminary and in need of replication against an attention placebo control condition (i.e., a group intervention of the same duration and format, but with content not related to addressing intersectional stigma). Further, recruiting eligible women in their first 28 weeks of pregnancy gave rise to pragmatic challenges that made strict randomization difficult. Instead, women’s allocation to the treatment and the control group was done systematically, albeit not in a true random fashion, via their recruitment timing. This pragmatic modification could have contributed to salient sociodemographic differences among women between the intervention arm (i.e., who were significantly more likely to not currently be in a long-term relationship; 55%) versus the TAU arm (20%; Table 2), and partner status could partially account for the elevated stigma and depression that we observed at baseline in the intervention group when compared with the control group. Nonetheless, we controlled for these baseline differences in depression and stigma in part through the use of a difference in difference analysis, which allowed us to control for baseline levels of variables by examining the pre-post difference for each individual. Moreover, scheduling conflicts made it difficult for pregnant mothers to attend all group stigma intervention sessions prior to delivery (i.e., weeks 36–40); we addressed this challenge by holding sessions twice a week when desired by and feasible for participants, but our small sample size precluded analyzing effects of this scheduling modification on outcomes. It was also unlikely for participants to be able to attend all sessions regardless of attempts to adjust scheduling; only 2 women were able to attend all 8 sessions. However, our intent-to-treat design meant we analyzed participants by their assigned intervention condition even if they did not attend all intervention sessions, which generated underestimates compared to if everyone had completed the intervention fully as intended. Furthermore, our results showing greater reductions in stigma and depression in the intervention arm (vs. TAU) despite the vast majority of women attending fewer than eight sessions suggests that our intervention could be shortened to less than eight sessions in future iterations without sacrificing effectiveness. Finally, given the MME intervention’s focus on WMM for pregnant women in Botswana, findings cannot be generalized to other groups; nonetheless, we assert that the WMM approach more generally can be applied to stigma measures and interventions and evaluated in new settings.

### Future directions

Our intervention’s intersectional, WMM-based approach and initial effectiveness results have implications for implementation in Botswana and for HIV stigma interventions elsewhere. First, while promising reductions in stigma and depression were observed, potential effects in improving ART adherence should be tested in a larger study using biological or other relatively objective measures. Measures of ART adherence should limit reliance on self-report measures, which are prone to social desirability and recall biases [21]. Using biological or other more objective measures in conjunction with self-reported ART adherence not only improves accuracy, but also would likely be more discriminant and clinically relevant in that they are more indicative of viral suppression and non-transmittable status to sexual partners and children [21–23]. Second, that mean gestational age was statistically higher in the intervention group when compared with the control group may warrant further investigation [16]. However, the mean gestational age in both groups is considered to be full-term and the observed difference in means is unlikely to have clinical significance. Additionally, even though the difference in birth weights between groups showed statistical significance, the lack of significant difference observed in weight-by-gestational-age z-scores suggests that this finding is due to the observed differences in gestational age. Third, the MME intervention is well-suited for embedding within routine ANC, which is free in Botswana. Finally, given prior findings that stigma manifests in ANC clinic-level practices among pregnant women with HIV in Botswana [4] and the desirability of intervening at the structural level to address intersectional stigma, targeting stigma at the healthcare facility level could augment effects via a multi-level intervention [7, 24, 25]. In closing, we found that identifying and targeting intersectional stigma via the WMM framework and promoting capabilities that ‘matter most’ for achieving ‘personhood’ facilitated a targeted HIV stigma intervention to show initial reductions in stigma and depression among WLHIV. We believe that the standardizable, WMM-based approach used to design this intervention can be applied and empirically tested to address other forms of intersectional stigma in new and diverse populations worldwide.

## Supplementary Information

**Additional file 1.** Overall characteristics of infants born to women enrolled in the Moving Mothers towards Empowerment Trial.

## Data Availability

The datasets used and/or analyzed during the current study are available from the corresponding author on reasonable request.

## Abbreviations

ANC: Antenatal care
ART: Antiretroviral therapy
IRB: Institutional Review Board
MME: Moving Mothers towards Empowerment
PMTCT: Prevention of mother to child transmission
SSA: Sub-Saharan Africa
TAU: Treatment as usual
WLHIV: Women living with HIV
WMM: What matters most
