# Supplementary material for: A pilot pragmatic trial of a “what matters most”-based intervention targeting intersectional stigma related to being pregnant and living with HIV in Botswana
Source: AIDS Res Ther. 2022 Jun 23;19:26. doi: 10.1186/s12981-022-00454-3 (PMC9219368; doi:10.1186/s12981-022-00454-3)
Supplement: Supplementary file 1 — Additional file 1. Overall characteristics of infants born to women enrolled in the Moving Mothers towards Empowerment Trial. [file 12981_2022_454_MOESM1_ESM.docx]

Additional file 1: Overall characteristics of infants born to women enrolled in the Moving Mothers towards Empowerment Trial

| **Measurement** | **Overall** | | |
| --- | --- | --- | --- |
|  | **n** | **Mean (SD) / %** | **Median** |
| **Male infants (sex documented at birth)** | 20 | 45.5 | - |
| **Birth weight (overall grams)** | 44 | 3147.0 | 3100.0 |
| *Males (grams)^* | 20 | 3119.5 | 3017.5 |
| *Females (grams)^* | 16 | 3217.8 | 3115 |
| *Sex not documented* | 8 | 3074.4 | 3005 |
| **Gestational age (weeks)** | 44 | 39.3 | 40.0 |
| *Males^* | 20 | 39.3 | 40.0 |
| *Females^* | 16 | 39.3 | 39.5 |
| *Sex not documented* | 8 | 39.4 | 39.5 |
| **Weight-for-gestational age (z-score)^^&^** | 36 | -0.25 | -0.21 |
| *Males^* | 20 | -0.45 | -0.26 |
| *Females^* | 16 | 0.00 | 0.12 |
| **APGAR 1 min** | 40 | 8.7 | 9.0 |
| *Males^* | 17 | 9.0 | 9.0 |
| *Females^* | 15 | 8.6 | 9.0 |
| *Sex not documented* | 8 | 8.4 | 8.5 |
| **APGAR 5 min** | 40 | 9.7 | 10.0 |
| *Males^* | 17 | 9.9 | 10.0 |
| *Females^* | 15 | 9.5 | 10.0 |
| *Sex not documented* | 8 | 9.4 | 9.5 |

Note. Data presented are on singletons only (i.e., two sets of twins were excluded from these analyses).

^ Among singletons with documented sex

^&^ Sex and birthweight standardized z-scores determined from study biometry using INTERGROWTH-21^st^ standards, accessed 8 May 2022 from URL: <http://intergrowth21.ndog.ox.ac.uk/>.
